# Spatial Characterization of Tumor Perfusion Properties from 3D DCE-US Perfusion Maps are Early Predictors of Cancer Treatment Response

**DOI:** 10.1038/s41598-020-63810-1

**Published:** 2020-04-24

**Authors:** Ahmed El Kaffas, Assaf Hoogi, Jianhua Zhou, Isabelle Durot, Huaijun Wang, Jarrett Rosenberg, Albert Tseng, Hersh Sagreiya, Alireza Akhbardeh, Daniel L. Rubin, Aya Kamaya, Dimitre Hristov, Jürgen K. Willmann

**Affiliations:** 1 Department of Radiology, Molecular Imaging Program at Stanford, School of Medicine, Stanford University, Stanford, CA USA; 2 Department of Radiology, Integrative Biomedical Imaging Informatics at Stanford, School of Medicine, Stanford University, Stanford, CA USA; 3 Department of Radiation Oncology, School of Medicine, Stanford University, Stanford, CA USA; 4 Department of Radiology, Body Imaging, Stanford University, Stanford, CA USA

**Keywords:** Tumour angiogenesis, Cancer imaging, Engineering

## Abstract

There is a need for noninvasive repeatable biomarkers to detect early cancer treatment response and spare non-responders unnecessary morbidities and costs. Here, we introduce three-dimensional (3D) dynamic contrast enhanced ultrasound (DCE-US) perfusion map characterization as inexpensive, bedside and longitudinal indicator of tumor perfusion for prediction of vascular changes and therapy response. More specifically, we developed computational tools to generate perfusion maps in 3D of tumor blood flow, and identified repeatable quantitative features to use in machine-learning models to capture subtle multi-parametric perfusion properties, including heterogeneity. Models were developed and trained in mice data and tested in a separate mouse cohort, as well as early validation clinical data consisting of patients receiving therapy for liver metastases. Models had excellent (ROC-AUC > 0.9) prediction of response in pre-clinical data, as well as proof-of-concept clinical data. Significant correlations with histological assessments of tumor vasculature were noted (Spearman R > 0.70) in pre-clinical data. Our approach can identify responders based on early perfusion changes, using perfusion properties correlated to gold-standard vascular properties.

## Introduction

Advances in anti-cancer agents have significantly enriched the therapeutic armamentarium available to clinicians for managing disease^1–3^, but have further complicated patient management because not all patients respond to treatments similarly^4^. There are currently no rapid and efficient methods to determine which treatment regimens will be effective on a patient-by-patient basis at baseline or within weeks of starting treatment. Conventional anatomical-based assessments with the Response Evaluation Criteria in Solid Tumors (RECIST 1.1) are performed at earliest 2-3 months after treatment start and do not account for acute cytostatic effects that do not always result in anatomical changes in lesion size^5^. Thus, there is a significant need for tools to rapidly assess or predict which patients will respond to treatments, with the potential to spare non-responding patients the high morbidity and cost associated with ineffective treatments.

Tumor vascular properties and perfusion before or during therapy are demonstrated predictive indicators of cancer response to several treatments^3,6–14^. Multiple minimally invasive functional perfusion imaging approaches are being explored to predict or monitor response to cancer therapies based on tissue perfusion and/or vascular properties. While many are very promising, radiation exposure (CT/PET), contrast restrictions due to potential adverse events (CT/MRI/PET), limited access (MRI/PET), high cost (MRI/PET), and inability to scan at the bedside are disadvantages for use in repetitive longitudinal exams. Dynamic contrast-enhanced ultrasound (DCE-US) is exempt from these limitations and offers non-invasive bedside functional imaging of tissue perfusion properties longitudinally^10,15–30^.

Prior studies have already demonstrated DCE-US’s potential in cancer treatment monitoring applications^31–39^. However, conventional use of DCE-US has to date been restricted to 2D ultrasound, with quantified perfusion parameters obtained as averages from a 2D region of interest (ROI) (i.e. conventional ROI-averaged parameters). This renders measurements prone to sampling errors and is unable to take into account the heterogeneous nature of tumor perfusion^40^. The recent commercial availability of matrix transducers with contrast-imaging mode has enabled three-dimensional (3D) DCE-US imaging in the clinic as a radiation-free and inexpensive bedside tool for longitudinal imaging to overcome sampling errors attributed to 2D-based imaging^41,42^. In addition, 3D DCE-US enables volumetric maps of perfusion parameters that can be used to characterize perfusion heterogeneities beyond conventional ROI-averaged parameters.

The use of quantitative histogram and texture features (henceforth image features) has been extensively used to characterize medical images, and more specifically, to capture the heterogeneity of tumor tissue-related parameters in medical imaging and radiomics^43–50^. It has been minimally explored in conventional 2D DCE-US in non-clinical imaging systems^51^. These image features can characterize spatial image properties beyond averaged image intensities, and are potentially more repeatable than standard intensity-based parameters. In this work, we sought to identify 3D DCE-US perfusion biomarkers on repeatable image features sensitive to early treatment-induced vascular changes and correlated to histology. We then used identified image features alone, or to develop multi-parametric machine-learning models to detect subtle perfusion characteristic changes in preclinical tumor tissues, and tested on a separate preclinical cohort, as well as proof-of-principle clinical data. Our study demonstrates that early perfusion characteristics and heterogeneity changes captured by image features/multi-parametric models, regardless of treatment or tumor type, predict treatment response.

## Results

### 3D DCE-US perfusion maps display longitudinal perfusion heterogeneity

**Figure 1:**
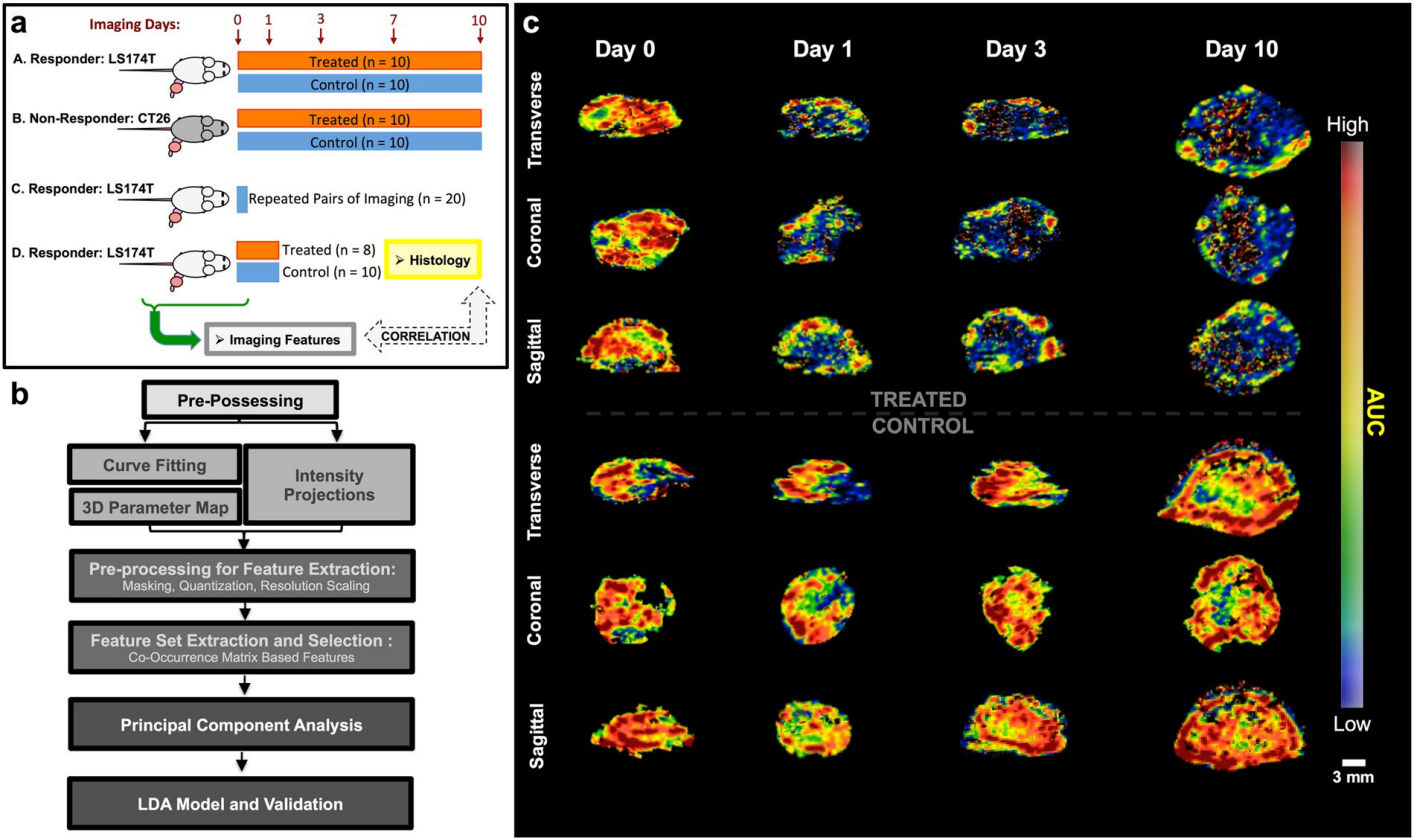
(**a**) Schematic of rodent data from LS174T human colon cancers (A, C, D) and CT26 rodent colon cancer (B). Treated animals received Bevacizumab at 10 mg/kg on days 0, 3 and 7. Data set A and B are longitudinally imaged and used as training in the PCA/LDA approach and the GLMNET approach. Data set C is a separate cohort of 20 tumors used for repeatability assessment. Data set D was used as test data acquired separately from the main cohort, and n = 11 out of the 18 animals (5 treated, 6 control) had whole tumor histological assessment of CD31 MVD at 24 hours after treatment. (**b**) Computational pipeline developed to generate parametric maps and extract features, PCA and develop the LDA model. Additional details on each of these steps is presented in supplementary methods. (**c**) Representative 3D maps of AUC. Note heterogeneous perfusion in baseline and longitudinally in treated/control groups.

We used a total of 78 mice bearing colon cancer tumors on the hind leg to identify multi-parametric biomarkers (cohorts A, B, C, D; detailed in Methods, Supplementary Methods and Fig. 1a). Animals were either implanted with human LS174T or mouse CT26 colon cancer cells and were either left as control or treated with the anti-angiogenic agent Bevacizumab; only LS174T cells are responsive to Bevacizumab. The different cohorts allowed us to develop models sensitive to perfusion characteristics based on image features (cohorts A and B), assess repeatability of image features (cohort C) and test our model in a separate data set with histology (cohort D). We applied a processing pipeline to extract 3D parametric maps of bolus-based tumor perfusion parameters from each data set on each imaging day (Fig. 1b)^52^. Briefly, this pipeline applies voxel-by-voxel fitting of a lognormal perfusion model to time-intensity curves (TIC) and generates a map for several bolus-based perfusion parameters (peak enhancement (PE), area under the curve (AUC), time to peak (TP), mean transit time (MTT) and bolus start time (T0)) using multi-core processing on a high-performance computing cluster. In addition to bolus-based perfusion parameters, 3 intensity projection volumetric images (maximum, average and standard deviation projection) were obtained from 4D data. Thus, for each 3D DCE-US data set, a total of 8 parametric maps and intensity projections (henceforth perfusion maps) were generated within a user-define volume of interest (VOI).

A representative perfusion map of the AUC is shown in Fig. 1c. Note heterogeneous perfusion parameter values throughout the tumor tissue longitudinally. In both control and treated mice, tumors developed minimally perfused regions in the lesion on day 10, with persisting heterogeneous flow throughout the rest of the lesion. A qualitative decrease in the average AUC was noted throughout the whole lesion within 24 hours of the first dose of Bevacizumab in treated animals; overall lesion perfusion remained heterogeneous. Longitudinally, non-perfused (necrotic) regions developed in most control and treated tumors on days 7-10.

### Features from parametric maps improve discrimination between tumor groups

**Figure 2:**
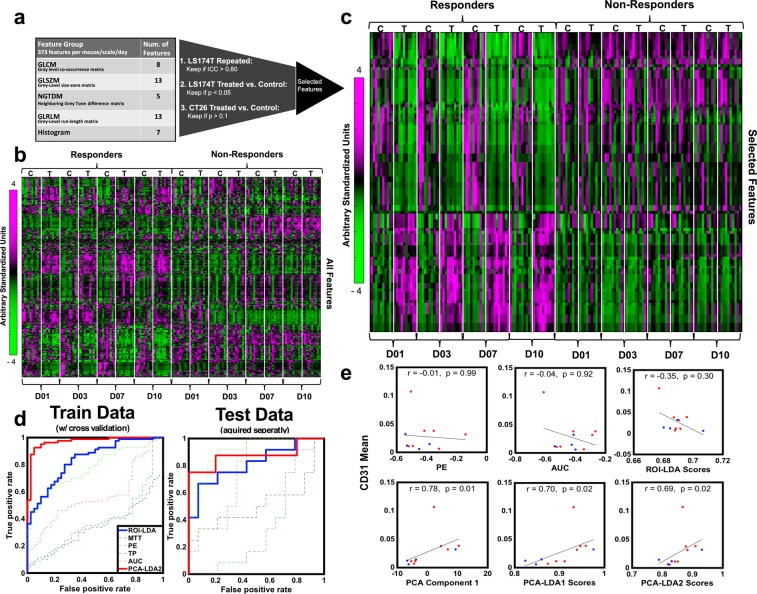
(**a**) Statistics-based feature selection process based on repeatability and sensitivity to treatment. (**b)** Heatmap showing all features (y-axis) on days 1, 3, 7 and 10 (D01-D10) – features are measured as percent change from baseline. Left is responder (LS174T) group and right is non-responder (CT26) group, with both treated (T) and control (C). (**c)** Same as D), but after feature selection. Note that within the responder group, there is an oscillation between treated (T) and control (C) animals from day 1 onwards, not observed in non-responder group. (**d)** ROC for conventional parameters alone (PE, AUC, TP, MTT – thin lines), combined in an LDA model (ROI-LDA; blue) and top 2 components from PCA in an LDA model (PCA-LDA2; red), in the training and test data set. (**e)** Correlations to histology of conventional parameters (top), LDA scores from conventional parameters (ROI-LDA; top), the first component from the PCA (bottom), and the LDA scores from top 16 PCA components (PCA-LDA1) and top 2 PCA components (PCA-LDA2) (bottom). These are shown as absolute measurements, and not percent change from baseline.

To compare image features extracted from 3D parametric maps to conventional ROI-averaged parameters, we extracted both and evaluated their performance in discriminating control and treated tumor groups exposed to Bevacizumab (Fig. 2a). A total of 1128 quantified image features were extracted and evaluated per animal on each imaging day and calculated as percent difference from baseline (pre-treatment). The different treatment evaluation days allowed us to evaluate different levels and a spectrum of treatment response (i.e. extent of change of vasculature and perfusion in lesion) relative to baseline. A complete list of all features is presented in Supplementary Table 1; a detailed description of features is available in supplementary methods. The average TIC obtained from a VOI was also used to extract conventional quantitative bolus perfusion parameters (henceforth termed conventional parameters) using a log-normal fit to the ROI-averaged bolus curve^33^. Conventional parameters are PE, AUC, TP and MTT; these were included in the complete set of 1128 features per animal/imaging day. To demonstrate that features from parametric maps can be sorted and combined to discriminate between tumor groups receiving Bevacizumab, we used two different methods; (i) a linear statistical approach and, (ii) a GLMNET approach.

#### Statistical approach

A statistics-based feature selection pipeline is presented in Fig. 2a and detailed in the methods section. This approach was chosen for its simplicity as a proof of concept. A heat map of all features is shown in Fig. 2b, from which no distinct patterns can be seen between treated and control tumors in either the responder group (LS174T) or the non-responder group (CT26). Overall, we selected a total of 90 features that are presented as a heat map in Fig. 2c and in Supplementary Table 2. Of the selected features, none were the conventional parameters PE, AUC, TP, or MTT. Note within the heat map (Fig. 2c) the alternating pattern between treated (T) and control (C) animals for the responder group that is not observed in the non-responder group.

We used a principal component analysis (PCA) for dimensionality reduction in order to eliminate redundant and/or correlated features and identify the dominant dimensions, reducing the total number of features available^53^. Ninety-eight percent of feature information was represented over 16 PCA components, while seventy percent of feature information was represented in 2 components. Overall, compared to conventional perfusion parameters (i.e. PE, AUC, TP, MTT), the two top dominant components performed better in discriminating between treated responders, and control or treated non-responders on a subject-by-subject basis to detect subtle vascular and perfusion changes, with a Receiver Operator Curve-Area Under the Curve (ROC-AUC) > 0.93 for each alone, in contrast to conventional parameters, which had a range of ROC-AUC of 0.40 – 0.75.

Two LDA models were generated based on PCA components, one was based on the top 16 PCA components (henceforth PCA-LDA1) and the other based on the top 2 PCA components (henceforth PCA-LDA2); these were generated using data from all of **Cohort A/B** over all treatment days and tested with 10-fold cross-validation and separately on **Cohort D**. Similarly, an LDA model based on the four main conventional ROI-averaged parameters (PE, AUC, TP, MTT) combined (henceforth ROI-LDA) was generated using the same data set and tested on **Cohort D**. ROC curves for the PCA-LDA2 and ROI-LDA models, along with ROC curves for each individual conventional parameter in **Cohort A/B (Train with cross-validation)** and **Cohort D (Separately acquired test data)**, are shown in Fig. 2d. While the ROI-LDA model had a ROC-AUC of 0.78 for **Cohort A/B** and 0.66 for the test **Cohort D**, the PCA-LDA2 model had a ROC-AUC of 0.96 for **Cohort A/B (Train with cross-validation)** and 0.88 for the test **Cohort D (Separately acquired test data)**. PCA-LDA1 model also performed well, with a ROC-AUC of 0.97 for **Cohort A/B** and 1 for the test **Cohort D;** select ROC-AUCs are summarized in Table 1.

**Table 1:** Area under curve (AUC) for ROC analysis to discriminate between responders and non-responders. CI is 95% confidence interval.

	Data Set A/B (Train Data)	Data Set D (Test Data)	Patients (Test Data)
GLMNET	—	0.95 (CI: 0.83, 1.00)	0.97 (CI: 0.82, 1.00)
PCA-LDA1	0.97 (CI: 0.93, 1.00)	1.00 (CI: 1,1)	0.94 (CI: 0.73, 1.00)
PCA-LDA2	0.96 (CI: 0.91, 1.00)	0.88 (CI: 0.77, 1.00)	0.99 (CI: 0.9, 1.00)
ROI-LDA (Conventional)	0.78 (CI: 0.68, 0.87)	0.66 (CI: 0.40, 0.92)	0.39 (CI: -0.01, 0.79)
PE-LDA	0.74 (CI: 0.64, 0.83)	0.76 (CI: 0.53, 0.99)	0.61 (CI: 0.19, 1.00)
AUC-LDA	0.43 (CI: 0.33, 0.52)	0.51 (CI: 0.23, 0.79)	0.28 (CI: -0.08, 0.64)
MTT-LDA	0.62 (CI: 0.51, 0.72)	0.65 (CI: 0.39, 0.91)	0.17 (CI: -0.11, 0.45)
TP-LDA	0.43 (CI: 0.33, 0.52)	0.38 (CI: 0.11, 0.64)	0 (CI: 0, 0)

#### GLMNET approach

We also tested a GLMNET-based approach to differentiate between responders and control/non-responders and train a model to detect subtle changes in perfusion charachteristics^54^. This approach was chosen due to its ability to handle high-dimensionality data. GLMNET uses a mixture of L2 and L1 regularization in fitting a generalized linear model, and as such, will “select features” by setting coefficients to 0 via the Lasso L1 component. Training of the GLMNET was done with Cohort A/B. All features were fed into the model as is to distinguish between responders and non-responders and to allow the model to select features using the complexity penalty method. We achieved consistent results across all tested alpha values, with ROC-AUC of 0.95 for mouse test data (**Cohort D**) and average ROC-AUC of 0.95 for human test data, indicating a well generalizing model.

### Biomarkers are correlated to histological quantification of vascular density

We tested whether selected PCA components and the scores from the PCA-LDA model from the statistical approach were correlated to volumetric histological characterization of mean vascular density (MVD) on CD31-stained tissue slides of the tumors. Parametric map image features were taken as absolute measurements to compare directly to histological measures, as opposed to change relative to baseline as per above, and were tested for correlation against volumetric histological evaluation of MVD at day 1 (24 hours after therapy) in **Cohort D**. The Spearman correlation coefficient was obtained between the MVD and each of the 16 PCA components and was found to correlate significantly (p < 0.05) to 10/16 components. The first component had the best correlation coefficient R (R = 0.78, p = 0.01), and is presented in Fig. 2e. The scores from the PCA-LDA1 and PCA-LDA2 models correlated with histology with an R of 0.70 and 0.69 (P = 0.02), respectively (Fig. 2e **bottom row**). In contrast, none of the conventional parameters alone, or combined in the ROI-LDA scores, correlated with histology (Fig. 2e **top row**).

### Multi-Parametric models based on features are predictive of early patient treatment response

**Figure 3:**
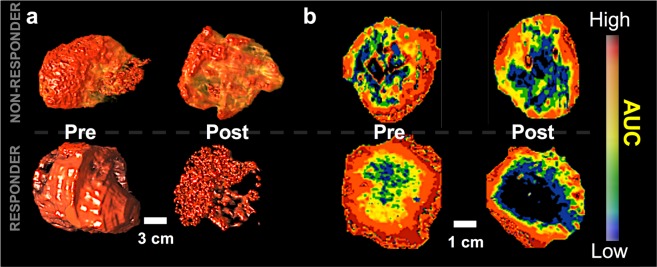
Representative 3D volumetric rendering of contrast signal **(a)**, and cross-section AUC parametric maps from responder and non-responder patients before and within 2 weeks after treatment **(b). (a)** 3D rendering of the AUC parametric map in the volume of interest (VOI). (**b)** Middle cross section of the VOI in the same order for both patients. Top row images are for patient 6 in Table 2 (Male, 52 y.o., responder), bottom row images are for patient 4 in Table 2 (Male, 68 y.o., non-responder). No significant changes in parametric map appearance is noted in non-responder.

We obtained human 3D DCE-US longitudinal bolus data acquired over 2 weeks (Day 0 before treatment and Day 14 after treatment start) in patients receiving cancer therapy as clinical proof-of- principle validation for the translational of our perfusion map measurements. All longitudinal data had a 60-day RECIST 1.1-based response evaluation as reference standard; responders were those reported with stable or regressed disease, and non-responders were those with progressive disease based on RECIST 1.1. All characteristics pertaining to acquired clinical data are summarized in Methods, Supplementary Methods, and Table 2. For each data set, eight parametric maps were generated using the same methods as described above, and image features were extracted for each parametric map to feed into the mouse-trained PCA-LDA1, PCA-LDA2, ROI-LDA and GLMNET models, as described above. Parametric maps for three representative patients are shown in Fig. 3. In the context of all patients, perfect discrimination was observed for PCA-LDA1 (AUC of ROC = 0.94). Similarly, we noted a ROC-AUC of 0.99 for PCA-LDA2. In contrast, a ROC-AUC of 0.39 for an LDA with all the conventional parameters was observed. For the GLMNET approach, the human data was used as test data with a non-responder\responder split of 3:4, indicating a well-balanced test set. With a model trained purely on mouse data, a human test set was able to achieve an AUC of 0.97.

**Table 2:** Longitudinal Early Clinical Validation Patient Data.

Primary Disease	Treatment	Response	Size	Age	Sex
Colorectal Adenocarcinoma	FOLFOX + Bevacizumab	Regression	9 cm	60	M
Pancreatic Neuroendocrine	Temozolomide + Capecitabine	Stable	2.4 cm	61	M
Pancreatic Adenocarcinoma	Capecitabine + Oxaliplatin	Stable	1.6 cm	54	M
Colorectal Adenocarcinoma	Irinotecan + Bevacizumab	Regression	4.3 cm	52	M
Pancreatic Adenocarcinoma	Capecitabine + Oxaliplatin	Progression	2.6 cm	70	F
Colorectal Adenocarcinoma	RRx-001	Progression	2.2 cm	68	M
Pancreatic Neuroendocrine	Everolimus + Octreotide + Embolization	Progression	9.5 cm	52	M
Pancreatic Neuroendocrine	Everolimus	Stable	2 cm	54	F
Pancreatic Adenocarcinoma	Gem-Abraxane	Stable	1.2 cm	72	F

## Discussion

In this study, we demonstrate the potential of 3D DCE-US multi-parametric models to predict early treatment response using quantitative image features extracted from perfusion maps. Our results indicate that image features can yield repeatable multi-parametric biomarkers alone or through machine learning models, and that are better than conventional parameters at detecting perfusion characteristics and heterogeneity; these can be used to discriminate between pre-clinical responders and non-responders in training and test preclinical data. Our early clinical validation data also suggests that these can be directly translated to the clinic to differentiate between human responders and non-responders within 14 days of treatment based on subtle tumor perfusion changes. Our study also demonstrates that multi-parametric biomarkers are significantly correlated to CD31 MVD from histology (p = 0.02-0.01).

Tumor vascular networks have direct implications for tumor progression and can directly influence tumor response to cancer therapies^14,55–57^. While several cancer therapies such as anti-angiogenics (i.e. Bevacizumab) directly target the process of angiogenesis, most other cancer therapies have indirect consequences on blood vessels and tumor blood flow, that in turn regulate treatment response, and remodel vascular networks in a way that can be predictive of treatment^23,58^. Studies have also demonstrated that perfusion changes following therapies are not uniform across the tumor tissue^59–65^. In contrast, most quantitative parameters that characterize tissue perfusion in medical imaging are based on averages from ROIs and ignore perfusion heterogeneities. In this work, we hypothesize that volumetric maps of perfusion parameters can reflect the heterogeneity of perfusion following therapy and that image features can capture heterogeneous perfusion changes.

The use of DCE-US in predicting or monitoring cancer therapy has received substantial attention in recent years as a potential tool to diminish healthcare costs and increase treatment efficacy^38^. This is due to inherent ultrasound advantages such as low cost, widespread availability and portability for longitudinal bedside imaging. While several DCE-US acquisition methods exist, bolus-based imaging and quantification is by far the most common method. Blood-volume based parameters (PE and AUC) have been previously correlated to treatment response^66,67^. Studies have also reported minimal to no correlation of bolus-based perfusion parameters to gold-standard histological assessments of vascular densities^41^. The current form of bolus-based DCE-US quantification, which evaluates parameters within a single ROI, assumes that this single averaged value is representative of the tissue examined. This may hold true in normal tissues, but is fundamentally flawed in heterogeneously perfused tumor tissues^38^. In addition, the 2D nature of ultrasound introduces significant potential for sampling errors that over or under-estimate perfusion parameters, which can be heavily skewed by the presence of a few large feeding fast-flow vessels within an ROI^38^.

The use of parametric maps to display perfusion maps has been explored in 2D DCE-US. Several strategies have been introduced to generate and qualitatively evaluate voxel-by-voxel parametric maps of perfusion parameters^68^. One study compared the histograms of flow speeds in renal cell cancers before and after an anti-angiogenic therapy and demonstrated that treatment predominantly affects the smallest vessels of the microvasculature^69^. However, to the best of our knowledge, with the exception of one recent study exploring radiomics on preclinical 2D contrast ultrasound data using a strictly preclinical imaging system for differentiating tumor type^51^, minimal quantitative strategies have been reported for predicting response from image features present within parametric maps. In addition, parametric maps of DCE-US parameters have only been generated in 2D. 3D DCE-US using clinical matrix transducers is a novel approach for treatment monitoring with enhanced repeatability of perfusion parameters^70^.

The extraction of quantitative 2D and 3D feature sets from radiological imaging data sets is a growing area of interest in radiology. Histogram and texture features from 2D images are amongst the most popular and reproducible^47,48,71,72^ because these focus on morphological and structural information within an ROI, as opposed to the subjective shape of an ROI. Several studies have reported on the use of texture features on ultrasound data, but to the best of our knowledge, no studies have performed texture analysis on 3D DCE-US perfusion maps. Our approach aims to statistically quantify patterns of perfusion through interconnected voxel patterns, as opposed to the intensity of contrast which can vary with the injection of the bolus and the number of microbubbles injected^73^. Thus, DCE-US perfusion maps potentially offer more repeatable quantification, which we did indeed confirm through this work.

Our results demonstrate that histogram and texture features from volumetric parametric maps maximize perfusion information beyond conventional averaged parameters and are better at differentiating between responders and non-responders. We were surprised to find high correlations for both individual PCA components and PCA-LDA1 scores to volumetric CD31 MVDs, especially given that conventional bolus parameters have been previously reported to not correlate with histology^41^. This supports that patterns captured through features are more representative of heterogeneous tumor perfusion than conventional parameters. Another important aspect of our study is the use of pre-clinical data to identify perfusion features that change in treated animals known to have real vascular alterations. Using these features, we developed models that capture tissue perfusion characteristic changes, which we found to translate to the clinic in pilot human data.

Future studies should address several limitations. First, the computational time it takes to form a parametric map is long due to the use of a high-level programing language such as Python. Future implementations in C/C + + or on a GPU could bring down the computational time to seconds. Second, the current frame rate in 3D contrast-mode is substantially lower than in 2D contrast-mode (1-5 Hz vs. 5-20 MHz). This can, in certain cases, affect the performance of fitting models used to obtain quantitative information, especially on a voxel-by-voxel basis. Although the frame rate is still good in comparison to most current DCE-MRI and DCE-CT methods, hemodynamic quantification would be superior if manufacturers improved on temporal resolution in 3D contrast-mode to match that of 2D contrast-mode. Third, assessment of reproducibility between different operators during data acquisition, and clinical repeatability of multi-parametric biomarkers, should be carried out to optimize data acquisition protocols and potentially further optimize our feature set. Finally, while our clinical data was appropriate for a first validation proof-of-principle translational testing of our methods, additional studies with a greater number of patients are warranted to confirm the potential of histogram and texture features in capturing volumetric perfusion information and predicting response in the clinic. This is also important for developing improved or more advanced models (i.e. neural networks) specifically trained *in human* data, as opposed to the simplistic models used in this paper to compare perfusion map measures vs. conventional parameters.

Our results set the stage for more precise quantification and characterization of perfusion from 3D DCE-US for treatment monitoring using a radiomics approach that includes machine learning, before anatomical changes are overtly visible based on current Response Evaluation Criteria in Solid Tumors (RECIST 1.1). Based on our work, further development of machine-learning models to detect subtle perfusion changes and predict responders based on improved feature-based quantification of perfusion patterns in volumetric data is promising. Beyond this, our work supports the notion that early perfusion attribute changes beyond the average perfusion intensity (i.e. blood volume) measured using image features in tumor tissues are predictive of treatment response. The bedside availability of 3D DCE-US can thus positively impact health care costs and provide rapid decision support in managing cancer patient treatment regimens.

## Methods

### Experimental design

Our experiments were designed to extract image features from 3D-DCE US longitudinal perfusion maps from liver metastases and to combine these as multi-parametric biomarkers that can detect subtle perfusion attribute changes and differentiate responders from non-responders. To identify reliable features from parametric maps, we isolated repeatable histogram and texture features (image features) to discriminate between responsive, control and non-responsive tumors treated with the anti-angiogenic agent Bevacizumab on a subject-by-subject basis. As a proof of concept, we investigated the use of two approaches to generate multi-parametric biomarkers for treatment assessment; i) a statistical approach, and ii) a GLMNET approach, developed on pre-clinical data and tested on pre-clinical test and early human proof-of-concept validation data. Pre-clinical test data consisted of Bevacizumab-treated and control animals, as well as a cohort for feature repeatability assessment. In addition, we tested in pre-clinical tissues whether multi-parametric biomarkers were correlated to volumetric histological quantification of vascular densities. Human data was obtained from ongoing larger trials to assess the feasibility of 3D DCE-US to monitor cancer therapy in patients with liver metastasis from the gastrointestinal (GI) tract; the data was used as initial pilot translational validation of 3D DCE-US parametric map-based biomarkers. Overall, we analyzed all available subjects (human and pre-clinical), applying stringent inclusion and exclusion criteria to homogenize our study cohorts as was most scientifically reasonable. All methods were performed in accordance with the relevant guidelines and regulations.

### Pre-Clinical data groups and measurements

All animal experiments were approved by the Stanford Administrative Panel on Laboratory Animal Care (APLAC), and were carried-out in accordance with the APLAC guidelines and regulations. Mice implanted with 20 tumors sensitive to Bevacizumab (LS174T human colon cancer; 10 control and 10 treated – **Cohort A**, Fig. 1a) and 20 tumors non-sensitive to Bevacizumab (CT26 murine colon cancer; 10 control and 10 treated - **Cohort B**, Fig. 1a) were imaged with 3D DCE-US on days 0, 1, 3, 7 and 10 following the start of a Bevacizumab treatment regimen (10 mg/kg on days 0, 3 and 7). Another 20 mice (**Cohort C**, Fig. 1a) implanted with the LS174T cell line (responsive to treatment) were imaged twice within one scan session to assess repeatability of quantitative parameters. Finally, a total of 18 mice with LS174T tumors responsive to treatment (**Cohort D**, Fig. 1a) were imaged at 24 hours to further test our biomarkers in a separate cohort of animals; 11 of these animals (5 treated and 6 control) had volumetric histology of CD31 mean vascular density (MVD) quantification to correlate to biomarkers. For all animals, imaging was carried out in 3D using a Philips EPIQ7 ultrasound machine coupled to a clinical X6-1 3D transducer using clinical grade contrast microbubble agents (Definity, Lantheus Medical Imaging, MA, USA) administered using the bolus DCE-US method. For the 20 LS174T animals in the repeatability group, two consecutive bolus data acquisitions were obtained during the same scan session, within 20 minutes of each other, to assess repeatability using the ICC. Additional details on the tumor model and image acquisition are provided in the supplementary methods.

### Conventional bolus DCE-US perfusion parameter extraction

All 3D DCE-US imaging datasets were analyzed using custom Python-based software (details in supplementary material). A VOI was manually contoured covering the whole tumor visualized on sagittal, longitudinal, and coronal planes in ITKsnap. A first-pass kinetics analysis of the average signal TIC from the VOI after bolus injection was used for the quantification of conventional tumor perfusion parameters from bolus 3D DCE-US^41^. Parameters included the PE and AUC, which are generally related to blood volume, and the TP and MTT, which are estimates of perfusion rates. Perfusion parameters were normalized to the baseline values (pre-treatment scan) to show percent changes.

### Parametric map generation

For all 3D DCE-US, a Python pipeline was developed to process images consistently in the same way in order to extract the bolus-based perfusion parameters on a voxel-by-voxel basis within the same pre-selected VOI used in conventional perfusion analysis. A total of 8 perfusion maps based on tracer model parameters and intensity projections were generated in 3D and saved as NIFTI files. Python-based software was used to generate perfusion maps using parallel processing on a high-performance multi-core processing computing cluster. It is comprised of 127-shared servers, which include 16 CPU cores per node, each operating with up to 4 GB of RAM. For parametric maps of tracer models (tens of millions of voxels present in each 3D perfusion image), the software ran in parallel on a single node for each data set, using all 16 cores per node (multi-processing) to parallelize voxel-by-voxel nonlinear least-square fitting of the lognormal model. Maps took an average of 3 hours to be generated. From this process, we obtained five 3D parametric maps and three 3D intensity projections. Detailed processing steps and pipelines are discussed in the supplementary methods. A total of 1128 features were obtained on each day, for each animal.

### Histogram and texture feature extraction

To conduct a proof-of-principle of model-based multi-parametric perfusion characterization, we evaluated two different linear machine learning modeling approaches.

#### Statistical approach

To reduce the number of features, we selected features that were: i) repeatable using the intra-class correlation coefficient (ICC > 0.8) using the repeatability data set (Fig. 1a**; Data Set C**), ii) that could significantly differentiate between the treated and control groups in mice with responding tumors at each imaging time point using a rank-sum test with a threshold of p < 0.05 (Fig. 1a**; Cohort A**), and iii) that did not differentiate between the treated and control animals in the treatment-resistant group, with a threshold of p > 0.1 (Fig. 1a**; Cohort B)**. Following feature selection, we used a principle component analysis (PCA) to isolate representative dimensions of selected features related to control/treated animals, eliminating redundancies (i.e. correlated features) and minimizing dimensionality in the feature set. An LDA model with 10-fold cross-validation was constructed based on the dominant dimensions, as per results.

#### GLMNET Approach

A GLMNET machine learning model was used to classify the data into responding or nonresponding groups. There was a total of 373 features. For the training data, **Cohort A/B were used**. For testing data, **Cohort D** was used. All features were evaluated as a relative change from baseline on each longitudinal scan day. Normalization across datasets was performed to account for widespread variations. Using ten-fold cross validation (CV), regularization parameter lambda was selected to minimize over-fitting and CV error for alpha values of 0.3, 0.5, 0.7, where alpha is the balance between L1 and L2 regularization. Features were selected by taking features with nonzero coefficients, and a ROC curve was computed for each alpha value, as well as the corresponding AUC.

### Patient data

Patient data was obtained from 9 patients with as clinical proof-of-concept validation of our approach from an ongoing HIPPA-compliant prospective study approved by the Stanford School of Medicine Institutional Review Board (IRB) and the Scientific Review Committee (SCR). All methods were performed in accordance with the relevant guidelines and regulations. Informed consent was obtained from all patients enrolled for 3D DCE-US imaging. Patients had at least one liver metastasis from a gastrointestinal or pancreatic primary tumor confirmed with MRI or CT. Full inclusion and exclusion criteria are presented in supplementary methods. For this purpose, imaging used for this proof-of-concept results consisted of two scans per patient within two weeks; the first scan right before treatment start and the second scan within 14 days $\pm$ 5 days of treatment start. At the end of the treatment cycle (around 60 days), treatment response was evaluated with an MRI/CT scan using “Response Evaluation Criteria in Solid Tumors (RECIST 1.1)” which is based on visible anatomical changes in size of the lesions^48^. Patients were then classified as responders or non-responders based on RECIST evaluation, and as described in supplementary materials.

### Statistical analysis and feature sorting

Statistical tests were used to evaluate repeatability and significance between different groups as indicated. To test for repeatability, the intra-class correlation coefficient (ICC) was used, where log-transformation was applied to normally distribute data for standard statistical analysis. Here, an ICC of 0-0.20 indicates no agreement; ICC of 0.21-0.40, poor agreement; ICC of 0.41-0.60, moderate agreement; ICC of 0.61-0.80, good agreement; and ICC greater than 0.80, excellent agreement. An unpaired Wilcoxon rank-sum test was used to compare the statistical significance of different groups with p < 0.05 indicating significance. Features were compared in different groups as absolute values, as well as relative changes (see **Supplementary Methods**). A sample size of 10 animals per group was chosen based on an estimation that it would provide 90% power with two-sided 5% error to detect differences as small as 1.5 standard deviations. The 95% confidence intervals (CI) were calculated in Prism (GraphPad, La Jolla, CA).

## Supplementary information

Supplementary Information.

## Data Availability

All data associated with this study are available in the main text or the supplementary materials.
