# Supplementary material for: Spatial Characterization of Tumor Perfusion Properties from 3D DCE-US Perfusion Maps are Early Predictors of Cancer Treatment Response
Source: Sci Rep. 2020 Apr 24;10:6996. doi: 10.1038/s41598-020-63810-1 (PMC7181711; doi:10.1038/s41598-020-63810-1)
Supplement: Supplementary file 1 — Supplementary Information. [file 41598_2020_63810_MOESM1_ESM.docx]

**TITLE:**

Spatial Characterization of Tumor Perfusion Properties from 3D DCE-US Perfusion Maps are Early Predictors of Cancer Treatment Response

**ONE SENTENCE SUMMARY:**

3D contrast ultrasound perfusion maps yield multi-parametric machine learning models that can longitudinally discriminate between responder and non-responder tumors.

**AUTHORS:**

Ahmed El Kaffas, PhD^1,2^, Assaf Hoogi, PhD^2^, Jianhua Zhou, MD^1^, Isabelle Durot, MD^1^, Huaijun Wang, MD PhD^1^, Jarrett Rosenberg, PhD^1^, Albert Tseng^1^, Hersh Sagreiya, MD^2^, Alireza Akhbardeh, PhD^1^, Daniel L. Rubin, MD^2^, Aya Kamaya, MD^1^, Dimitre Hristov, PhD^3^, Jürgen K. Willmann, MD^1^

**AFFILIATIONS:**

^1^Department of Radiology, Molecular Imaging Program at Stanford, School of Medicine, Stanford University, Stanford, CA

^2^Department of Radiology, Integrative Biomedical Imaging Informatics at Stanford, School of Medicine, Stanford University, Stanford, California

^3^Department of Radiation Oncology, School of Medicine, Stanford University, Stanford, CA

**CORRESPONDING AUTHOR:**

Ahmed El Kaffas, Ph.D.

Department of Radiology

School of Medicine, Stanford University

1201 Welch Road, Rm P097, Stanford, CA 94305

P: 650-353-6495; Fax: 650-723-1909

Email: elkaffas@stanford.edu

**SUPPLEMENTARY METHODS**

**Colon Cancer Xenograft Model of Responder and Non-Responder: Description, Experimentation and Validation**

All experimental procedures involving laboratory animals were approved by the Institutional Administrative Panel on Laboratory Animal Care. A total of 78 female nude mice (Charles River, Wilmington, MA; 6-8 weeks old; 20-25 g) were induced with human (LS174T; ATCC, Manassas, VA; n=58) or murine (CT26; ATCC, Manassas, VA; n=20) colon cancer. The human colon tumor line actively responds to clinical (humanized) VEGF-targeting therapy such as Bevacizumab, while the murine line is resistant to such treatments. Together, these two cell lines can be used to simulate clinical responders and non-responders to anti-angiogenic therapy, with the CT26 cell line acting as a negative control^1,2^. From the n=58 mice bearing LS174T tumors, 20 were used for control (n=10) and treatment (n=10), 20 were used for repeatability assessment and 18 were used as a separate test data set imaged only at baseline and 24 hours. Of the 18 animals used as test data, 11 had histology at 24 hours for correlation of perfusion features with CD31. Animals bearing CT26 were used as control (n=10) and treatment (n=10) non-responders (negative-control) to Bevacizumab. A complete schematic of the experimental design and animal genotypes is presented in **Figure 1A**. Both cell lines were grown in Dulbecco’s Modified Eagle Medium (DMEM; Gibco, Grand Island, NY), supplemented with 10% fetal bovine serum (Gibco), penicillin (50 U/ml), and streptomycin (50 µg/ml) at 37°C in a humidified 5% CO2 atmosphere. At 70%-80% confluence, the tumor cells were collected following trypsinization, and 4×10^6^ LS174T cells or 1×10^6^ CT26 cells suspended in 50μl phosphate-buffered saline and 50μl Matrigel (BD Biosciences, San Jose, CA) were injected subcutaneously on the right lower hind limb of the nude mice. Both LS174T and CT26 tumors were allowed to grow for 10 days to an average diameter of 10 mm (range: 6-14 mm) in the maximum direction (measured with electronic caliper). Treatment responses were previously confirmed using tumor growth and immunohistology ^2^. For ***histological analysis*** of tumor vasculature, tumors were surgically removed and fixed in 4% paraformaldehyde and PBS solution for 24h, sectioned into 10 μm slices for CD31 immunofluorescence staining using standard methods ^1–3^. A subset of tumors had several sections cut as previously described to sample volumetric vascular density for correlation ^4^. Fluorescent microscopy was performed by using a LSM510 metaconfocal microscope (Zeiss, Maple Grove,MN) and a high-resolution digital camera (AxioCam MRc, Bernried, Germany) under 200-fold magnification. The mean vascular density (MVD) per slice was quantified by using Image J software (National Institutes of Health, Bethesda, MD) as the average value from 5 randomly selected fields of view (single field of view area, 0.19 mm^2^). The MVD was significantly (*p* <0.001) lower in bevacizumab-treated compared to control responder tumors (LS174T) [REF]. No significant differences (*p* > 0.05) in the MVD between treated and control tumors were noted in non-responders (CT26) [REF]. ***Tumor growth*** was monitored by using electronic calipers measurements available on the ultrasound system using the following formula: volume = π/6 × *L* × *W* × *H*, where *L* is length, *W* is width, and *H* is height. Measurements were significantly different between control and treated responders (LS174T) on day 3, 7 and 10 (p<0.001), but not on day 1 or on any day in non-responders (CT26) (p > 0.05)^2^.

**Antiangiogenic Treatment**

Mice were randomized into groups as per Figure 1A. Treated animals received the anti-angiogenic Bevacizumab (Avastin, Genentech, South San Francisco, CA) at baseline (immediately after imaging) as well as on days 3 and 7 (in animals followed beyond 24 hours) after the first injection of the drug. The agent was diluted in sterile saline to a dose of 10 mg/kg b.w. (corresponding to a fluid volume of 10 μl/g b.w.) and administered intravenously. The same volume of saline alone was administered on the same days in control animals. A total of 11 animals in the treatment (n=6) and control (n=5) groups for LS174T that were imaged and sacrificed at baseline and 24 hours following the start of therapy only, with the goal of characterizing early manifestations of vascular density.

**Three-Dimensional DCE-US Data Acquisition**

DCE-US uses FDA-approved intravascular ultrasound contrast agents, which consist of highly echogenic, micron-sized gas bubbles (microbubbles), stabilized by a shell made from biodegradable materials ^5–7^. These enable qualitative tissue perfusion assessment, as well as quantitative parameterization of tissue blood flow and blood volume^8^.

Imaging was carried out in 3D using a Philips EPIQ7 coupled to a clinical X6-1 3D transducer using clinical grade contrast microbubble agents (Definity, Lantheus Medical Imaging, MA, USA)<sup>4,9</sup><i>(4, 9)</i> administered using the bolus DCE-US method. Definity microbubbles (FDA-approved perfluoro microbubbles) were diluted 1:4 in sterile 0.9% saline after activation as per manufacturer. A suspension of 120 μL was administered over 5 seconds (at a constant injection rate of 24 μl/sec) with an infusion pump. For the 20 LS174T animals in the repeatability group, two consecutive bolus data acquisitions were obtained during the same scan session. The second bolus was administered ~15 minutes after the first bolus to ensure that the contrast was cleared from the systemic flow of the animal to eliminate signal interference with the second bolus injection signal. For both LS174T and CT26 animals, control and treated tumors were imaged at baseline and at 1, 3, 7, and 10 days following the start of therapy. For each bolus, 3D DCE-US datasets were acquired in real time over four minutes in each mouse by using a built-in Digital Navigation Link of the ultrasound system to custom in-house MevisLab modules written in C++^10–13^.

For all imaging procedures, mice were anesthetized with inhalation of 2% isoflurane in room air (administered at 2 L/min). Mice were placed prone on a heated imaging stage in order to maintain constant body temperature for the entire duration of the experiments. A 27G needle catheter (Vevo Micromarker; VisualSonics, Toronto, Canada), which attached to an infusion pump (Kent Scientific, Torrington, CT), was placed into one of the two tail veins for contrast agent injection. The transducer was held in a fixed position with a clamp to minimize motion artifacts. To reduce artifacts in the near-field zone of the clinical transducer, a customized standoff ultrasound gel was placed on the skin of all mice. The distance between the transducer and the center of the tumor was set at 3 cm. The following imaging parameters were kept constant for all 3D DCE-US imaging experiments in all tumors: center frequency, 3.2 MHz; mechanical index, 0.09; volume rate, 1 Hz; dynamic range, 52 dB; focus placed beneath the tumor (4-6 cm from transducer head).

**Conventional Bolus DCE-US Perfusion Parameter Extraction**

All 3D DCE-US imaging datasets were analyzed using custom Python-based software (available at https://github.com/aelkaffas/3DDCEUSParametricMaps). To generate volumes of interest (VOI), we manually contoured the whole tumor visualized on sagittal, longitudinal, and coronal using ITKsnap to produce volumes of interest.

Image analysis was performed by one reader with 6 years of DCE-US experience in random order. The reader was blinded to the treatment information and types of animals (LS174T vs. CT26). All 3D DCE-US imaging datasets were analyzed with a custom software in MeVisLab as previously described ^4^. A first-pass kinetics analysis of the signal intensity-time curve from the VOI, which is based on the wash-in and washout kinetics of microbubbles after bolus injection, was used for the quantification of tumor perfusion. For conventional parameter extraction, the average of the linearized intensities of all the pixels in the VOI was used to obtain a time-intensity curve. A lognormal model was fitted to each perfusion curve to extract the following perfusion parameters: peak enhancement (PE, arbitrary units, au), area under the curve (AUC, au), mean transit time (MTT, seconds) and time to peak (TP, seconds). The PE and AUC are generally related to blood volume while the TP and MTT are estimates of perfusion rates^14^. Perfusion parameters on days 1, 3, 7 and 10 were normalized to the baseline values to show percent changes.

**Parametric Map Generation**

A total of 8 perfusion maps based on tracer model parameters and intensity projections were generated in 3D and saved as NIFTI files. Python-based software was used to generate perfusion maps using parallel processing on a high-performance multi-core processing computing cluster. The cluster is used for voxel-by-voxel least-square fitting of time-intensity curves to an established perfusion model for parametric map generation. It is comprised of 127-shared servers, which include 16 CPU cores per node, each operating with up to 4 GB of RAM. For parametric maps of tracer models (tens of millions of voxels present in each 3D perfusion image), the software ran in parallel on a single node for each data set, using all 16 cores per node to perform a voxel-by-voxel nonlinear least-square fitting of the lognormal model. Maps take an average of 3 hours to be generated. Unlike CT and MRI data, ultrasound images are noisy and filed with artifacts. To account for this, several ‘cleanup’ steps were applied to subtract major artifacts in images in the 4D sequence, and to smoothen the time-intensity curves. More specifically, the processing pipeline included: **(1)** linearization of the signal and isotropic resampling to generate a time intensity curve (TIC); **(2)** identification of contrast arrival in image (wash-in start) and auto-masking of flow-only voxels (perfused tissue); **(3)** standardizing TIC fitting (which also check for quality of fit) to each voxel based on the log-normal perfusion model or an intensity projection; and **(4)** generation of parametric maps or intensity projection (**Figure 1B**). Additional parametric maps of different parameters are displayed in **Supplementary Figure 1**. Parametric maps include: peak enhancement (PE), area under the curve (AUC), time-to-peak (TP), mean transit time (MTT), arrival time of contrast (T0), maximum intensity projection (MIP), average intensity projection (AIP), and standard deviation projection (SIP). For clinical data, an additional motion correction step was employed ^15,16^. This is necessary given increased motion in clinical data compared to pre-clinical data.

**Histogram and Texture Feature Extraction**

Radiomics-based image features can generally be obtained from any image, and can broadly divided into low-level (i.e., histogram) and high-level (i.e., non-texture and texture) features ^17–21^. Low-level features are based on pixel statistics, from histogram intensity-based parameters (mean, median, and skewness of grey levels). Non-texture features are based on segmented lesions such as tumor size/volume (longest diameter, short axis, etc.) and tumor shape (circularity, compactness, etc.). While histogram features capture information regarding the spread and shape of image histograms (such as mean, median, and skewness of grey levels, etc), texture features take into account the overall statistical relationship of one voxel to another with the aim of capturing intensity patterns and heterogeneities with a single quantified value ^17,18,28–37,19,21–27^. This goes beyond basic averages of voxel intensities, and is especially advantageous to focus the quantification on the patterns of perfusion, as opposed to intensities of contrast, which may be affected by attenuation or contrast preparation or administration. These have been shown to be good descriptors of the heterogeneity of tumor tissue or perfusion, and sensitive indicators of treatment response in various imaging modalities ^17,18,28–32,38–42,19,43–45,21–27^.

Histogram and texture features were extracted from each perfusion and intensity projection map from 3D DCE-US data, for each animal or patient data on each scan day. For histogram features, the mean, median, mode, standard deviation, skewness and kurtosis of the intensity distribution were extracted. For texture features, all parametric maps were quantified exactly the same by: i) confining the ROI to the smallest box/region possible in image to speed up the computation, ii) normalization, iii) isotropic resampling of pixels, iv) quantizing of pixels (lloyd quantization) (**Figure 1B**) ^46,47^. Features were extracted at the following 3 length scales by resampling the isotropic voxel size to the following dimension: 0.3, 0.6 and 0.9 mm, in all possible directions. Four different categories of second-order texture features were extracted as described in **Supplementary Table 1.** These include the Grey Level Co-Occurrence Matrix, Grey Level Size-Zone Metric, Grey Level Run-Length Matrix and Neighborhood Grey Tone Difference Matrix.

For each animal and at each time point, a total of 1128 features were extracted over all three length scales and all parametric maps/intensity projections. Additional details and the code used to extract parametric maps and features are included in the supplementary data of this text. All features and conventional parameters were evaluated as relative change (*(X­_D01_ – X_D00_)/X_D00_; (X­_D03_ – X_D00_)/X_D00_; X­_D07_ – X_D00_)/X_D00_; X­_D10_ – X_D00_)/X_D00_*), except when correlated to histopathology, where absolute measurements of features were obtained on data acquired on the day of animal sacrifice for histology. Features were selected as described and are presented in **Supplementary Table 2**.

**Patient Data**

Patient data from ongoing HIPPA compliant prospective clinical studies at our institution (approved by the Institutional Review Board of our institution and written consent was obtained from all participating patients) using 3D DCE-US was used as pilot proof-of-concept preliminary data for this study to determine if treatment response could be predicted based on perfusion changes assessed with 3D DCE-US radiomics in patients with metastatic liver cancer receiving treatment. For this purpose, data from 9 adults were used. Inclusion criteria were: provide written consent, willing to comply with protocol, at least 18 years of age or older, and at least one liver metastasis from a gastrointestinal or pancreatic primary tumor confirmed with MRI or CT. A clinical oncologist referred each patient after introducing the study to the patient. Exclusion criteria were: documented anaphylactic or other severe reaction to any contrast media; pregnant or lactating patients; and patients with cardiac shunts or presence of severe pulmonary hypertension. Exclusions are based on contraindication for ultrasound contrast agent. No patient was excluded due to the exclusion criteria. Included patients are summarized in Table 2. Alll patients were scanned on Day 0 before initiating treatment, and within 14 days $\pm$ 5 days after therapy start. At the end of the treatment cycle (around 60 days), treatment response was evaluated with an MRI/CT scan using “Response Evaluation Criteria in Solid Tumors (RECIST 1.1)” which is based on visible anatomical changes in size of the lesions ^48^. Briefly, the 3D DCE US scanned liver metastasis, among other tumor relevant lesions in the liver and/or other organs, was identified in the baseline CT scan before treatment start as “target lesion” and the size of all target lesions was again evaluated in the follow-up scan. The sum of diameter of all target lesions was assessed and treatment response classified as “Progressive Disease” (>+20% or new lesion), “Stable Disease”(<+20% to -30%), “Partial Response”(>-30%), or “Complete Response”(all target lesions disappeared). Based on that, patients with “Progressive Disease” were categorized in the current study as non-responder (n=3), and the rest as treatment responders (n=4).

**SUPPLEMENTARY RESULTS**

**Classification rule for LDA model for 2 PCA components (PCA-LDA2):**

$$Y= (1.89)+(0.05)\times X1+(-0.05)\times X2$$

SUPPLEMENTARY TABLES

Supplementary Table 1: Summary of texture features

| **Type** | **Description and Reference** | **Features** |
| --- | --- | --- |
| **Global features (first-order gray-level statistics)**  Standard first order histogram-derived features. |  | Mean  Median  Mode  Standard Deviation  Variance (1)  Skewness  Kurtosis  Entropy (1) |
| **Gray-Level Co-occurrence Matrix (GLCM)**  Most commonly used feature sets; uses a co-occurrence matrix looking at frequency of co-occurrence between two pixels using a set directionality and scale. | Haralick *et al* 1973. | Energy  Contrast (1)  Correlation  Homogeneity  Variance (2)  Sum Average  Entropy (2)  Dissimilarity |
| **Gray-Level Run-Length Matrix (GLRLM)**  The matrix is based on the run-length of voxels with same grey levels given a specific direction. | Galloway 1975 | Short Run Emphasis (SRE)  Long Run Emphasis (LRE)  Gray-Level Non-uniformity (GLN) (1)  Run-Length Non-uniformity (RLN)  Run Percentage (RP) |
|  | Chu *et al* 1990 | Low Gray-Level Run Emphasis (LGRE)  High Gray-Level Run Emphasis (HGRE) |
|  | Dasarathy and Holder 1991 | Short Run Low Gray-Level Emphasis (SRLGE)  Short Run High Gray-Level Emphasis (SRHGE)  Long Run Low Gray-Level Emphasis (LRLGE)  Long Run High Gray-Level Emphasis (LRHGE) |
|  | Thibault *et al* 2009 | Gray-Level Variance (GLV) (1)  Run-Length Variance (RLV) |
| **Gray-Level Size Zone Matrix (GLSZM)**  This matrix is independent of direction, and is built on the GLRLM methods as foundation. It estimates the size of zones within an image that have the same grey levels. | Galloway 1975, Thibault *et al* 2009 | Small Zone Emphasis (SZE)  Large Zone Emphasis (LZE)  Gray-Level Non-uniformity (GLN) (2)  Zone-Size Non-uniformity (ZSN)  Zone Percentage (ZP) |
|  | Chu *et al* 1990, Thibault *et al* 2009 | Low Gray-Level Zone Emphasis (LGZE)  High Gray-Level Zone Emphasis (HGZE) |
|  | Dasarathy and Holder 1991, Thibault *et al* 2009 | Small Zone Low Gray-Level Emphasis (SZLGE)  Small Zone High Gray-Level Emphasis (SZHGE)  Large Zone Low Gray-Level Emphasis (LZLGE)  Large Zone High Gray-Level Emphasis (LZHGE) |
|  | Thibault *et al* 2009 | Gray-Level Variance (GLV) (2)  Zone-Size Variance (ZSV) |
| **Neighborhood Gray-Tone Difference Matrix (NGTDM)**  Aims to capture the grey scale difference between a pixel and neighboring pixels. Features have been shown to capture true nature of texture in images similar to human perception. | Amadasun and King 1989 | Coarseness  Contrast (2)  Busyness  Complexity  Strength |

Supplementary Table 2 - List of Selected Features: ‘(Parametric Map)-(Feature)’

| Selected Features  (Length Scale = 0.3 mm) | | Selected Features  (Length Scale = 0.6 mm) | Selected Features  (Length Scale = 0.9 mm) |
| --- | --- | --- | --- |
| 'AIP-Energy' | 'SIP-ZSV' | 'AIP-RLV' | 'AIP-Skewness' |
| 'AIP-Variance (1)’ | 'SIP-LRE' | 'AIP-Skewness' | 'AIP-Kurtosis' |
| 'AIP-LGZE' | 'SIP-GLN (2)' | 'AIP-Kurtosis' | 'MIP-Sum Average' |
| 'AIP-HGZE' | 'SIP-HGRE' | 'MIP-RLV' | 'MIP-GLN (2)' |
| 'AIP-SZLGE' | 'SIP-SRHGE' | 'MIP-Variance (2)' | 'MIP-RLV' |
| 'AIP-SZHGE' | 'SIP-LRLGE' | 'SIP-Variance (1)' | 'MIP-Variance (2)' |
| 'AIP-LZLGE' | 'SIP-LRHGE' | 'SIP-RLV' | 'SIP-Contrast (1)' |
| 'AIP-LRE' | 'SIP-RLV' | 'SIP-Variance (2)' | 'SIP-Variance (1)' |
| 'AIP-GLN (2)' | 'SIP-Variance (2)' | 'PE-Energy' | 'SIP-SRHGE' |
| 'AIP-LRLGE' | 'PE-LRLGE' | 'PE-Variance (1)' | 'SIP-RLV' |
| 'AIP-RLV' | 'PE-RLV' | 'PE-ZSV' | 'SIP-Contrast (2)' |
| 'AIP-Skewness' | 'AUC-Energy' | 'PE-GLN (2)' | 'SIP-Variance (2)' |
| 'AIP-Kurtosis' | 'AUC-ZSN' | 'AUC-Energy' | 'SIP-Skewness' |
| 'MIP-Variance (1)' | 'AUC-SZHGE' | 'AUC-SZE' | 'AUC-Energy' |
| 'MIP-LZLGE' | 'AUC-LRE' | 'AUC-ZSN' | 'AUC-SZE' |
| 'MIP-LRE' | 'AUC-GLN (2)' | 'AUC-LRE' | 'AUC-LZE' |
| 'MIP-GLN (2)' | 'AUC-LGRE' | 'AUC-GLN (2)' | 'AUC-ZSN' |
| 'MIP-HGRE' | 'AUC-SRLGE' | 'AUC-RLV' | 'AUC-LZHGE' |
| 'MIP-SRHGE' | 'AUC-LRLGE' | 'PE-Energy' |  |
| 'MIP-LRHGE' | 'AUC-RLV' | 'PE-Variance (1)' |  |
| 'MIP-Busyness' | 'TP-ZP' |  |  |
| 'MIP-Variance (2)' | 'T0-LZLGE' |  |  |
| 'SIP-Energy' |  |  |  |
| 'SIP-Variance (1)' |  |  |  |
| 'SIP-LZE' |  |  |  |
| 'SIP-GLN (1)' |  |  |  |
| 'SIP-HGZE' |  |  |  |
| 'SIP-SZHGE' |  |  |  |
| 'SIP-LZLGE' |  |  |  |
| 'AIP-Energy' |  |  |  |

**SUPPLEMENTARY REFERENCES**

1. Zhou, J. *et al.* VEGFR2-Targeted Three-Dimensional Ultrasound Imaging Can Predict Responses to Antiangiogenic Therapy in Preclinical Models of Colon Cancer. *Cancer Res.* **76**, 4081–9 (2016).

2. Zhou, J. *et al.* Early prediction of tumor response to bevacizumab treatment in murine colon cancer models using three-dimensional dynamic contrast-enhanced ultrasound imaging. *Angiogenesis* **20**, 547–555 (2017).

3. Obermueller, E., Vosseler, S., Fusenig, N. E. & Mueller, M. M. *Cooperative Autocrine and Paracrine Functions of Granulocyte Colony-Stimulating Factor and Granulocyte-Macrophage Colony-Stimulating Factor in the Progression of Skin Carcinoma Cells*. (2004).

4. Wang, H., Hristov, D., Qin, J., Tian, L. & Willmann, J. K. Three-dimensional Dynamic Contrast-enhanced US Imaging for Early Antiangiogenic Treatment Assessment in a Mouse Colon Cancer Model. *Radiology* **277**, 424–34 (2015).

5. Dijkmans, P. a *et al.* Microbubbles and ultrasound: from diagnosis to therapy. *Eur. J. Echocardiogr.* **5**, 245–56 (2004).

6. Stride, E. Physical principles of microbubbles for ultrasound imaging and therapy. *Cerebrovasc. Dis.* **27 Suppl 2**, 1–13 (2009).

7. William D. O’Brien. Ultrasound—biophysics mechanisms. *Prog Biophys Mol Biol. 2007* **93**, 212–255 (2007).

8. Sadeghi-Naini, A. *et al.* Imaging innovations for cancer therapy response monitoring. *Imaging Med.* **4**, 311–327 (2012).

9. Wang, H., Kaneko, O. F., Tian, L., Hristov, D. & Willmann, J. K. Three-dimensional ultrasound molecular imaging of angiogenesis in colon cancer using a clinical matrix array ultrasound transducer. *Invest. Radiol.* **50**, 322–9 (2015).

10. Schlosser, J. *et al.* Robotic intrafractional US guidance for liver SABR: System design, beam avoidance, and clinical imaging. *Med. Phys.* **43**, 5951–5963 (2016).

11. Wang, H., Lutz, A. M., Hristov, D., Tian, L. & Willmann, J. K. Intra-Animal Comparison between Three-dimensional Molecularly Targeted US and Three-dimensional Dynamic Contrast-enhanced US for Early Antiangiogenic Treatment Assessment in Colon Cancer. *Radiology* **282**, 443–452 (2017).

12. El Kaffas, A. *et al.* Quantitative Three-Dimensional Dynamic Contrast-Enhanced Ultrasound Imaging: First-In-Human Pilot Study in Patients with Liver Metastases. *Theranostics* **7**, 3745–3758 (2017).

13. Schlosser, J., Kirmizibayrak, C., Shamdasani, V., Metz, S. & Hristov, D. Automatic 3D ultrasound calibration for image guided therapy using intramodality image registration. *Phys. Med. Biol.* **58**, 7481–96 (2013).

14. Strouthos, C., Lampaskis, M., Sboros, V., McNeilly, A. & Averkiou, M. Indicator dilution models for the quantification of microvascular blood flow with bolus administration of ultrasound contrast agents. *IEEE Trans. Ultrason. Ferroelectr. Freq. Control* **57**, 1296–310 (2010).

15. El Kaffas, A. *et al.* Novel Motion Correction Algorithm for 3D Dynamic Contrast Ultrasound Without Anatomical Bmode Images. in *IEEE IUS 2018* (2018).

16. Chen, J.-S.-, Goubran, M., Kim, G., Willmann, J. K. & El Kaffas, A. Motion Correction of 3D Dynamic Contrast-Enhanced Ultrasound Imaging without Anatomical Bmode Images. *Submitt. to IEEE TUFFC* (2019).

17. Wu, C. M., Chen, Y. C. & Hsieh, K. S. Texture features for classification of ultrasonic liver images. *IEEE Trans. Med. Imaging* **11**, 141–52 (1992).

18. Felipe, J. C., Traina, A. J. M. & Traina, C. Retrieval by content of medical images using texture for tissue identification. in *16th IEEE Symposium Computer-Based Medical Systems, 2003. Proceedings.* 175–180 (IEEE). doi:10.1109/CBMS.2003.1212785

19. Gao, S. *et al.* Texture analysis and classification of ultrasound liver images. *Biomed. Mater. Eng.* **24**, 1209–16 (2014).

20. Amadasun, M. & King, R. Textural features corresponding to textural properties. *IEEE Trans. Syst. Man Cybern.* **19**, 1264–1273 (1989).

21. Chen, D.-R. *et al.* Classification of breast ultrasound images using fractal feature. *Clin. Imaging* **29**, 235–45 (2005).

22. Alic, L., Niessen, W. J. & Veenland, J. F. Quantification of heterogeneity as a biomarker in tumor imaging: A systematic review. *PLoS One* **9**, 1–15 (2014).

23. O’Connor, J. P. B. *et al.* Imaging intratumor heterogeneity: Role in therapy response, resistance, and clinical outcome. *Clin. Cancer Res.* **21**, 249–257 (2015).

24. Kothari, S., Phan, J. H., Stokes, T. H. & Wang, M. D. Pathology imaging informatics for quantitative analysis of whole-slide images. *J. Am. Med. Inform. Assoc.* **20**, 1099–108 (2013).

25. Gangeh, M. J., Kaffas, A. El, Hashim, A., Giles, A. & Czarnota, G. J. Advanced machine learning and textural methods in monitoring cell death using quantitative ultrasound spectroscopy. in *2015 IEEE 12th International Symposium on Biomedical Imaging (ISBI)* 646–650 (IEEE, 2015). doi:10.1109/ISBI.2015.7163956

26. Kather, J. N. *et al.* Multi-class texture analysis in colorectal cancer histology. *Sci. Rep.* **6**, 27988 (2016).

27. Ahmed, A., Gibbs, P., Pickles, M. & Turnbull, L. Texture analysis in assessment and prediction of chemotherapy response in breast cancer. *J. Magn. Reson. Imaging* **38**, 89–101 (2013).

28. Rose, C. J. *et al.* Quantifying spatial heterogeneity in dynamic contrast-enhanced MRI parameter maps. *Magn. Reson. Med.* **62**, 488–499 (2009).

29. Ng, F., Kozarski, R., Ganeshan, B. & Goh, V. Assessment of tumor heterogeneity by CT texture analysis: Can the largest cross-sectional area be used as an alternative to whole tumor analysis? *Eur. J. Radiol.* **82**, 342–348 (2013).

30. Ng, F., Ganeshan, B., Kozarski, R., Miles, K. A. & Goh, V. Assessment of Primary Colorectal Cancer Heterogeneity by Using Whole-Tumor Texture Analysis: Contrast-enhanced CT Texture as a Biomarker of 5-year Survival. *Radiology* **266**, 177–184 (2013).

31. Chicklore, S. *et al.* Quantifying tumour heterogeneity in 18F-FDG PET/CT imaging by texture analysis. *European Journal of Nuclear Medicine and Molecular Imaging* **40**, 133–140 (2013).

32. Amadasun, M. & King, R. Textural features corresponding to textural properties. *IEEE Trans. Syst. Man. Cybern.* **19**, 1264–1274 (1989).

33. Livens, S. *et al.* A Texture Analysis Approach to Corrosion Image Classification. *Microsc. Microanal. Microstruct.* **7**, 143–152 (1996).

34. Materka, A. & Strzelecki, M. Texture Analysis Methods – A Review. 1–33 (1998).

35. Arivazhagan, S. & Ganesan, L. Texture classification using wavelet transform. *Pattern Recognit. Lett.* **24**, 1513–1521 (2003).

36. Vos, W. H. De. *Focus on Bio-Image Informatics*. **219**, (Springer International Publishing, 2016).

37. Vallières, M., Freeman, C. R., Skamene, S. R. & El Naqa, I. A radiomics model from joint FDG-PET and MRI texture features for the prediction of lung metastases in soft-tissue sarcomas of the extremities; SUPPLEMENTARY INFORMATION. *Phys. Med. Biol.* **60**, 5471–5496 (2015).

38. Wu, W.-J. & Moon, W. K. Ultrasound breast tumor image computer-aided diagnosis with texture and morphological features. *Acad. Radiol.* **15**, 873–80 (2008).

39. Depeursinge, A., Kurtz, C., Beaulieu, C., Napel, S. & Rubin, D. Predicting visual semantic descriptive terms from radiological image data: preliminary results with liver lesions in CT. *IEEE Trans. Med. Imaging* **33**, 1669–76 (2014).

40. Depeursinge, A. *et al.* Automated classification of usual interstitial pneumonia using regional volumetric texture analysis in high-resolution computed tomography. *Invest. Radiol.* **50**, 261–7 (2015).

41. Itakura, H. *et al.* Magnetic resonance image features identify glioblastoma phenotypic subtypes with distinct molecular pathway activities. *Sci. Transl. Med.* **7**, 303ra138 (2015).

42. Nicolasjilwan, M. *et al.* Addition of MR imaging features and genetic biomarkers strengthens glioblastoma survival prediction in TCGA patients. *J. Neuroradiol.* **42**, 212–21 (2015).

43. Depeursinge, A. *et al.* Optimized steerable wavelets for texture analysis of lung tissue in 3-D CT: Classification of usual interstitial pneumonia. in *2015 IEEE 12th International Symposium on Biomedical Imaging (ISBI)* 403–406 (IEEE, 2015). doi:10.1109/ISBI.2015.7163897

44. El Kaffas, A. *et al.* Development of First In Human 3D Parametric Maps for Texture Analysis of Volumetric DCE-US to Spatially Monitor Flow Patterns During Colorectal Cancer Liver Metastases Treatment. in *World Molecular Imaging Conference* (2015).

45. El Kaffas, A. *et al.* Quantitative Image Features from 3D Contrast-Enhanced Ultrasound Parametric Maps as Surrogate of Treatment Response. in *World Molecular Imaging Conference* (2016).

46. Lloyd, S. Least squares quantization in PCM. *IEEE Trans. Inf. Theory* **28**, 129–137 (1982).

47. Collewet, G., Strzelecki, M. & Mariette, F. Influence of MRI acquisition protocols and image intensity normalization methods on texture classification. *Magn. Reson. Imaging* **22**, 81–91 (2004).

48. Eisenhauer, E. A. *et al.* New response evaluation criteria in solid tumours: Revised RECIST guideline (version 1.1). *Eur. J. Cancer* **45**, 228–247 (2009).
